# Isolation and Identification of Bone Marrow Mesenchymal Stem Cells from Forest Musk Deer

**DOI:** 10.3390/ani13010017

**Published:** 2022-12-20

**Authors:** Weiqiang Luo, Yangyang Geng, Mengxi Gao, Mengting Cao, Junjian Wang, Jing Yang, Chenxuan Sun, Xingrong Yan

**Affiliations:** Shaanxi Key Laboratory for Animal Conservation, College of Life Sciences, Northwest University, Xi’an 710069, China

**Keywords:** bone marrow, differentiation, forest musk deer, mesenchymal stem cells, RNA sequencing

## Abstract

**Simple Summary:**

The forest musk deer is a wild animal that is endangered due to widespread hunting for its musk, which is used in medicine and perfume. Stem cells can differentiate into a variety of cells, tissues, organs, and even complete individuals, which can be used for cell therapy and germplasm preservation. Mesenchymal stem cells are the most abundant stem cells in animals and have been isolated from many animals, such as cows and dogs. In this study, to evaluate the feasibility of isolating mesenchymal stem cells from forest musk deer, bone marrow was collected from forest musk deer, and the cells were isolated and characterized. They exhibit the same stemness and differentiation capacity as other mammals studied previously. Therefore, bone marrow mesenchymal stem cells may be an important tool for forest musk deer cell therapy.

**Abstract:**

The forest musk deer (Moschus berezovskii) is an endangered animal that produces musk that is utilized for medical applications worldwide, and this species primarily lives in China. Animal-derived musk can be employed as an important ingredient in Chinese medicine. To investigate the properties of bone marrow mesenchymal stem cells (MSCs) obtained from the bone marrow of forest deer for future application, MSCs were isolated and cultivated in vitro. The properties and differentiation of these cells were assessed at the cellular and gene levels. The results show that 81,533 expressed genes were detected by RNA sequencing, and marker genes of MSCs were expressed in the cells. Karyotype analysis of the cells determined the karyotype to be normal, and marker proteins of MSCs were observed to be expressed in the cell membranes. Cells were differentiated into osteoblasts, adipocytes, and chondroblasts. The expression of genes related to osteoblasts, adipocytes, and chondroblasts was observed to be increased. The results of this study demonstrate that the properties of the cells isolated from bone marrow were in keeping with the characteristics of MSCs, providing a possible basis for future research.

## 1. Introduction

The forest musk deer, also known as *Moschus berezovskii*, is a small-sized and artiodactyl animal primarily observed in southwestern Asia [1]. Forest musk deer can produce musk as a raw material for Chinese medicine, such as Angong Niuhuang Wan and Pian Zai Huang [2,3]. Therefore, in nature, the number of forest musk deer has decreased due to overhunting and habitat destruction. Forest musk deer is listed in class I of key wildlife species by the Chinese Wild Animal Protection Law, the Appendices of the Convention on the International Trade of Endangered Species of Wild Fauna and Flora [1,4]. To maintain ecological equilibrium and protect the resources of traditional Chinese medicine, wildlife forest musk deer were protected by hunting prohibition and habitat protection. Furthermore, some forest musk deer were caged under the Chinese Wild Animal Protection Law to meet the demands of traditional Chinese medicine for musk.

Mesenchymal stem cells (MSCs) are the most abundant cell population in the body. They exist in many tissues and have extensive self-renewal capacity and the ability to differentiate into multiple lineages [5,6]. MSCs comprise several lineages, such as bone marrow MSCs (BM-MSCs), adipose MSCs, and umbilical cord blood MSCs [7]. MSCs regulate immune tolerance in allogeneic transplantation [8]. MSCs derived from rat bone marrow have the chronic potential to differentiate into osteoblasts, adipocytes, and neural cells [9]. Since MSCs have a strong proliferation and differentiation ability, they can be used as donor cells of somatic cell nuclear transfer (SCNT) for cloning animals [10]. Moreover, MSCs can also differentiate into germ cells by co-culture with Sertoli cells [11]. Therefore, MSCs are among the seed cells applied to cell therapy, endangered animal protection, and animal husbandry.

There are few reports about MSCs’ application in endangered animals. MSCs were isolated from giant panda bone marrow and identified at the cellular and molecular levels [12]. MSCs from the bone marrow of red pandas were also characterized and were similar to those from other species, exhibiting such properties as the expression of multiple marker genes during differentiation [13].

The forest musk deer is an endangered animal species. To date, there has been no published research on the MSCs from this species. Therefore, in-depth study of the characteristics and functions of MSCs is beneficial for the protection of the forest musk deer. In this study, MSCs were isolated from the bone marrow of forest musk deer and were characterized by cell morphology, surface markers, and differentiation ability. The results of this study may provide seed cells for cell therapy and potential clinical application.

## 2. Materials and Methods

### 2.1. Reagents

All reagents were purchased from Sigma Aldrich (Shanghai, China) unless stated otherwise.

### 2.2. Animal

Forest musk deer were caged in Feng County, Baoji city, Shaanxi Province. The experiments performed in this study were approved by the Animal Ethics Committee of Northwest University in China. A 3-year-old male forest deer that had died of dyspnea caused by pulmonary infection with *Pseudomonas aeruginosa* was analyzed. The thigh bone was isolated sterilely by surgery and sent to the laboratory in 4 °C PBS with 2% penicillin-streptomycin.

### 2.3. Isolation and Culture of Cell from Bone Marrow

This method was the same as that described in a previous report [14]. Briefly, the marrow was aspirated aseptically by a trocar connected to a 10 mL syringe. After suction, the marrow was placed into a 10 cm plate, and 10 mL of DMEM with 20% fetal bovine serum (FBS) was added to the marrow cells, which were incubated for 7 d in an incubator at 37 °C, 5% CO_2_ and in saturated humidity. Subcultivation was performed as cell growth reached 80% confluence. Cells were digested with 0.25% trypsin and 0.04% EDTA, neutralized in DMEM with 10% FBS, and washed at 1000 rpm centrifugation for 3 min. The supernatant was discarded, and a single-cell suspension was performed by the addition of fresh medium. The concentration of cells was established as 1 × 10^6^/mL, and a 1 mL suspension was placed in a 10 cm plate with 9 mL DMEM with 10% FBS; Next, the cells were incubated to 80% confluence in an incubator.

### 2.4. Karyotype Analysis of Cells

The karyotype analysis was performed using a protocol described in a previous report [15]. Briefly, cells at a concentration of 5 × 10^5^ /mL were cultured in 10 cm plates. After 36 h, cells were treated with colchicine (0.3 µg/mL) for 6 h. Next, cells were digested with 0.25% trypsin combined with 0.04% EDTA, followed by treatment with hypotonic solution (0.075 M KCl), and the cells were fixed with fixation solution (methane: acetic acid = 3:1) three times. Cell fixation solution was added dropwise to slides to a height of more than 10 cm. After air drying, cells were stained with staining solution (Giemsa:PBS = 1:9). The chromosomes were observed and photographed under a microscope. The karyotype was subsequently constructed by arranging the sizes of the chromosomes.

### 2.5. Flow Cytometry

Flow cytometry was performed according to a protocol described in a previous report [16]. Briefly, cells were digested by trypsin and neutralized in medium with 10% FBS and rinsed three times with PBS. A single-cell suspension was made by pipetting cells up and down in PBS. Cells were diluted to a concentration of 1 × 10^6^ cells/mL. Cells were fixed with 2% polyformaldehyde (PFA) for 20 min and washed three times with PBS. Cells were labeled with FITC-conjugated antibodies against CD34, CD166, CD90, CD14, CD 44, CD45, CD71, CD105 and CD29 (Table 1). FITC-conjugated mouse IgG1 was used as the isotype-matched control. Cells were analyzed by FACS flow cytometry using Cell Quest software.

### 2.6. Differentiation of MSCs

For adipogenic differentiation, preformation was performed according to a protocol described in a previous report [17]. Cells at passage 3 were digested, and a suspension of single cells was made. A total of 8 × 10^4^ cells were seeded in a 3.5 cm plate and cultured for 24 h; consequently, the medium was replaced by adipogenic differentiation medium (DMEM containing 10% FBS, 500 μM isobutylmethylxanthine, 1 μM dexamethasone, 10 μM insulin, and 200 μM indomethacin). The medium was changed twice a week. After 21 d, the cells were rinsed twice with PBS and fixed with 4% PFA for 10 min. The cells were stained with staining solution (60% isopropanol containing 0.3% Oil Red) for 20 min and washed twice with 60% ethanol. The cells were observed under an inverted microscope.

For osteogenic differentiation, cells were digested with 0.25% trypsin and washed with PBS. Cells (5 × 10^4^) were placed in 3.5 cm plates and cultured for 24 h. The culture medium was replaced by aesthetic differentiation medium (DMEM containing 0.1 μM dexamethasone, 300 μM ascorbic acid, and 10 mM β-glycerophosphate) for 3 weeks [18]. The medium was changed twice a week. Cells were washed with PBS twice and fixed with 4% PFA. Cells were stained with Alizarin Red. After washing with PBS twice, the cells were observed under an inverted microscope.

For chondrogenic differentiation, 1 × 10^6^ MSCs at passage 3 were seeded in a 3.5 cm plate. After 24 h, chondrogenic medium (DMEM containing 0.1 μM dexamethasone, 25 μM ascorbic acid 2-phosphate, and 1 ng/mL TGF-β) was added. The medium was changed twice a week. After 21 d, the cells were washed twice with PBS and fixed with 4% PFA. The cells were stained with Alcian Blue and observed under an inverted microscope.

### 2.7. RNA Sequencing of Cells

Cells at 3 passages were grown to 80% confluence and were washed twice with PBS. The cells were lysed by adding TRIzol reagent. Total RNA was extracted by a mirVana miRNA Isolation Kit (Ambion-1561, Ambion Inc., Austin, TX, USA) following the manufacturer’s protocol, and RNA quality was evaluated by a NanoDrop 2000. RNA was reverted to cDNA by PrimeScript 1st Strand cDNA Synthesis Kit (D6110A, TaKaRa, Beijing, China), cDNA was fragmented with an Agencourt AMPure Kit (A63881, Beckman Coulter, Inc., Brea, CA, USA), and paired-end 150 bp fragments were sequenced by HiSeqTM 2500 instrument. Raw data were screened by Perl script, by which adapters, redundant reads, and low-quality data were removed. Clean data were evaluated, including data quality and GC content. The data were mapped to a eukaryotic reference genome. GO analysis was performed by Blast2GO [19]. GO and KEGG terms were analyzed, and the CDS was predicted.

### 2.8. Extraction of Total RNA and qPCR

Cells at 3 passages undergoing adipogenic and osteogenic differentiation in 3.5 cm plates were washed 3 times. Total RNA was extracted by TRIzol reagent, isopropanol, and ethanol. cDNA synthesis was performed by a PrimeScript 1st Strand cDNA Synthesis Kit (D6110A, TaKaRa). qPCR analysis was performed using a PCR mix, and the primers designed by Primer 5.0 based on the predicted CDS (Table S1). qPCR was performed according to the following cycling protocol: 95 °C for 5 min, 45 cycles of 95 °C for 5 s, and 58 °C for 10 s. The threshold cycle (Ct) value was normalized to β-actin. ΔΔCt was used to calculate the differential expression of genes.

### 2.9. Analysis

All experiments were performed in triplicate, and data were analyzed by SPSS 13.0. The data are shown as means ± standard deviation (SD). Comparative analysis was performed by paired *t*-test, and *p*-values < 0.05 were considered to indicate significant differences.

## 3. Results

### 3.1. Morphology and Proliferation of MSCs

**Figure 1:**
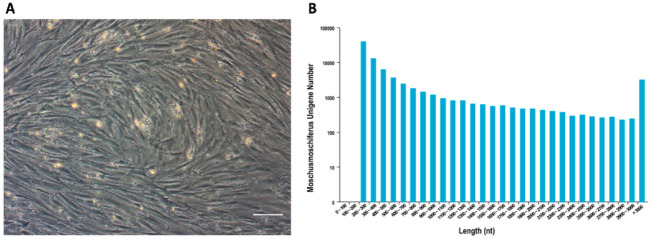
(**A**) Cell morphology of MSCs in vitro cultured at 3 passages. Cells exhibited spindle and fusiform shapes. Scale bar: 100 μm; (**B**) Unigenes length distribution after transcriptome sequencing and de novo assemble. The x-axis represents the length of unigenes, and the y-axis represents the number of unigenes within a certain length range.

After incubation of MSCs for 48 h, the cells growing adherently under the sparse hemocytes were visualized by shaking the culture dishes. At first, the morphology varied, with some cells appearing spindle-shaped and others round. On day 3 of culture, colonies had formed. On day 7, cells had overspread to the bottom of the culture dishes, and most cells were spindle-shaped. At passage 3, forest musk deer MSCs exhibited the characteristic fibroblastic MSC morphology, similar to that observed for rodents and humans (Figure 1A). Cells at passage 9 still showed a strong proliferation ability.

### 3.2. RNA Data Were Detected by RNA Sequencing

Passage 3 MSCs’ mRNA was extracted and sequenced. A total of 49,943 unigenes were spliced in more than 100 bp, and most genes were distributed in the range of 200 bp to 300 bp (Figure 1B). A total of 23,666 unigenes were annotated by nr, Pfam, GO, and KEGG, which included cell surface markers (e.g., *CD105*, *CD106*, *CD73*, *CD44*, and *CD29*), unveiling a list of candidate markers that could provide a basis for antibody-mediated immunophenotyping by flowcytometry and sequences for gene expression quantification by qRT-PCR.

### 3.3. Normal Karyotype was Maintained in MSCs

**Figure 2:**
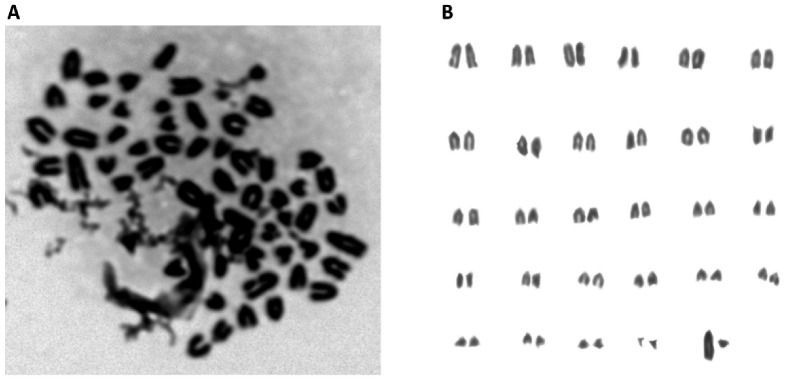
(**A**) Karyotype analysis of MSCs. Chromosomes were obtained from MSCs at M phase; (**B**) Karyotype analysis showed a normal karyotype. Fifty-eight chromosomes were revealed, including the XY sex chromosomes.

As shown in Figure 2, chromosomes in the M phase were collected from MSCs (Figure 2A), and the total number was 58 (Figure 2B). Additionally, after the chromosomes were aligned, the karyotype was observed to be normal. The sex chromosomes were XY, indicating that the sample was derived from forest musk deer. These results demonstrate that the karyotype was normal.

### 3.4. Surface Markers Were Detected by Flowcytometry

**Figure 3:**
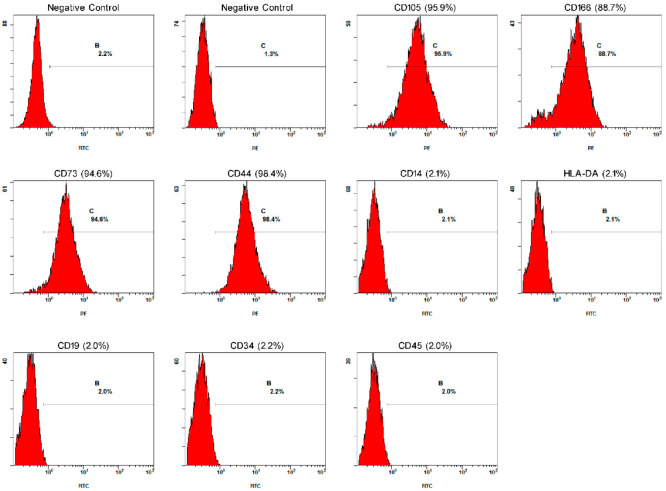
MSC expressed marker genes. Negative control without fluorescence staining of FITC and PE. The expression of CD105, CD166, CD73, CD44, CD14, HLA-DR, CD19, CD34, and CD45 in MSCs at 3 passages was detected by FACS. MSCs positively expressed CD105, CD166, CD73, CD44, but negatively expressed CD14, HLA-DA, CD19, CD34, CD45. B and C in the FACS panels represent FITC and PE-positive cells, respectively.

To characterize the MSCs, immunophenotyping and immunofluorescence techniques were applied. The immunophenotypic profiles of the positive values obtained for CD105 (95.9%), CD166 (88.7%), CD73 (94.6%), CD44 (98.4%), CD14 (2.1%), HLA-DA (2.1%), CD19 (2.0%), CD34 (2.2%), and CD45 (2.0%) are shown in Figure 3. The immunofluorescence technique obtained positive results for CD105, CD73, CD166, and CD44 and negative results for CD14, HLA-DA, CD19, CD34, and CD45. The cells exhibited membrane markers similar to those of MSCs.

### 3.5. Differentiation Ability

**Figure 4:**
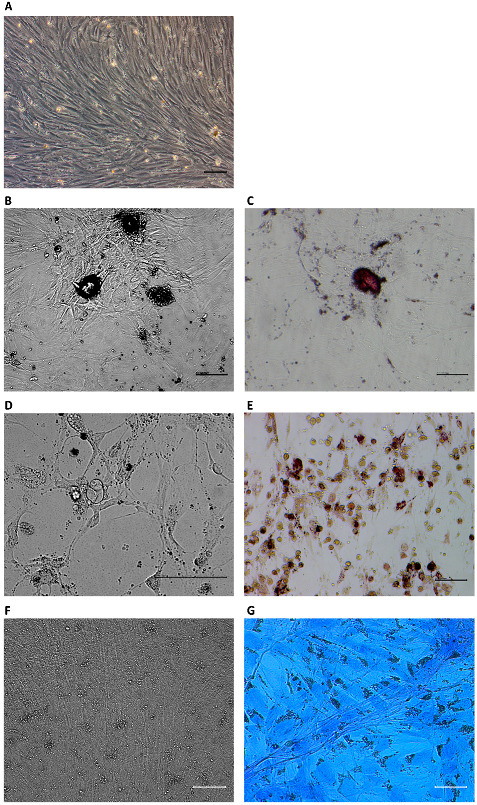
Multilineage differentiation potential of MSCs at P4. Cells before differentiation (**A**). Osteoblasts derived from MSCs (**B**) were stained with Alizarin Red (**C**). Adipocytes derived from MSCs (**D**) were stained with Oil Red (**E**). Chondrogenic derived from MSCs (**F**) contained proteoglycans stained with Alcian Blue (**G**). Scale bar: 100 μm.

MSCs have the potential to differentiate into osteoblasts in vitro. After treatment with osteogenic differentiation medium, the growth of cells gradually slowed and eventually stopped. Cell morphological changes were observed over the period of treatment. The cells showed polygonal morphology after culture in the medium for 21 d (Figure 4B). Bone extracellular matrix deposition was observed by Alizarin Red staining (Figure 4C). For adipogenic differentiation, the growth of cells gradually decreased and stopped over the period of exposure to adipogenic differentiation medium. Cell morphology was changed with treatment in adipogenic differentiation medium for 21 d, the volume of cells became larger, and lipid vacuoles accumulated in the cytoplasm (Figure 4D). Cytoplasmic lipids were observed by staining with Oil Red (Figure 4E). For chondrogenic differentiation, after 21 days of culture, the cells were aggregated and separated by the extracellular matrix (Figure 4F). Proteoglycans were observed by staining with Alcian Blue (Figure 4G). These results demonstrated that MSCs could differentiate into osteoblasts, adipocytes, and chondrocytes.

### 3.6. Expression Level of mRNA Decreased after Differentiation

**Figure 5:**
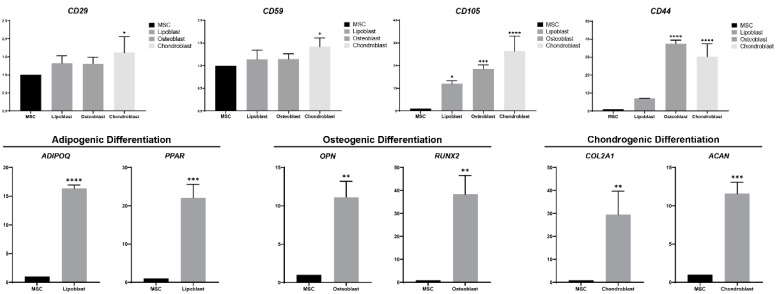
Gene expression of MSCs by differentiating into lipoblasts, osteoblasts, and chondroblasts. Marker gene expression (*CD29*, *CD59*, *CD105*, and *CD44*) of MSCs after differentiation; marker gene expression of adipocytes (*ADIPOQ* and *PPAR*), osteoblasts (*OPN* and *RUNX2*) and chondroblasts (*COL2A1* and *ACAN*) after the differentiation of MSCs. * *p* < 0.05, ** *p* < 0.01, *** *p* < 0.001, **** *p* < 0.0001.

To identify the gene expression profile after differentiation, the expression levels of genes in osteoblasts, adipocytes, and chondroblasts were compared with those of MSCs (Figure 5). *CD29* and *CD59* were expressed in MSCs, the expression of which was higher after differentiation, but no significant difference was observed in lipoblasts and osteoblasts (*p* > 0.05). *CD105* expressed at higher levels in three differentiated cells. *CD44* with a higher expression in three differentiated cells, but no significance was observed in lipoblasts (*p*>0.05). The expression of *OPN* and *RUNX2* was significantly upregulated in osteoblasts. *ADIPOQ* and *PPAR* were highly expressed in adipocytes. The expression of *COL2A1* and *ACAN* was significantly increased compared to undifferentiated MSCs. These results suggest that the expression profile changed due to the differentiation of MSCs into adipocytes, osteoblasts, and chondroblasts.

## 4. Discussion

MSCs are stem cells that can differentiate into other lineages. MSCs can be obtained from bone marrow and other organs and tissues, and these cells are non-hematopoietic stem cells with multipotential differentiation [5]. Meanwhile, MSCs can be employed in cell therapy by secreting many cell factors to assist in restoring damaged tissue [20]. MSCs are also applied as donors for cloning animals [21]. Therefore, MSCs are among the seed cells that may be stored for future applications. For endangered animals, MSCs are an important genetic resource. MSCs have been isolated and characterized from various species, including humans [5], experimental animals such as mice [22], rats [9], and rabbits [23], and endangered animals such as giant pandas [12] and red pandas [13].

In this study, we successfully isolated MSCs from the bone marrow of forest musk deer. The MSCs appeared to have a typical fibroblast-like shape associated with other MSCs [7,12,13,24,25,26]. MSCs were originally isolated from bone marrow [5], but isolation in other tissues such as adipose tissue [27,28], umbilical cord blood [29], and placenta [30] have been reported. Adipose tissue can be obtained by a less invasive method and in larger quantities than bone marrow and exhibits a similar morphology, immunophenotype, and differentiation potential [31]. However, we isolated MSCs from bone marrow because the lower level of fat is stored in the forest musk deer, and it is difficult to separate.

Karyotype analysis confirmed that MSCs contain 58 chromosomes, which is consistent with the normal karyotype of forest musk deer reported by Chi et al. [32]. We sequenced the transcriptome to acquire MSC marker gene cDNA sequences of forest musk deer because of the lack of a reference genome for *M. berezovskii*, and 49,943 and 23,666 unigenes were assembled and annotated, respectively.

Since no specific antibodies against forest musk deer are commercially available at present, we used antibodies against human CD105, CD166, CD73, CD44, CD14, HLA-DA, CD19, CD34, and CD45 to analyze MSCs by flow cytometry, as these markers are conserved across species based on RNA-seq data and the same antibody has been used to label markers in rats, rabbits, and common marmosets [33,34,35,36]. According to the International Society of Cellular Therapy (ISCT), MSCs must have positive expression of CD44, CD90, and CD105 and lack expression of CD14, CD34, CD45, and HLA-DA surface antigens [37]. The results of FACS confirmed the presence of MSC surface markers (CD105+/CD44+/CD166+) and the absence of CD14, CD19, CD34, CD45, and HLA-DA, similar to MSCs from other species [9,22,38].

MSCs have the ability for multi-differentiation potential, including differentiation into osteoblasts, adipocytes, chondrocytes [5], muscle cells [39], and neurons [40]. In this study, MSCs derived from bone marrow were observed to differentiate into osteoblasts, lipoblasts, and chondroblasts. The RNA-seq data were used for the design of primers used for RT-PCR in this study. CD44 is the major cell-surface receptor for hyaluron and is generally strongly expressed in osteoblasts and chondroblasts [41,42], which was observed in our research. Intense expression of CD105 was observed in osteoblasts and chondroblasts, which resonates with a previous study showing that CD105 is important for MSC chondrogenic differentiation [43]. CD59 is a key complementary regulatory protein that inhibits the formation of the membrane attack complex (MAC), which promotes inflammatory and degradation in chondroblasts [44]. CD29, also known as integrin β1, mediates the binding of chondrocytes to extracellular matrix proteins [45]. We examined the expression level of the osteogenic, adipogenic, and chondrogenic differentiation marker genes *OPN, RUNX2*, *ADIPOQ*, *PPAR*, *COL2A1,* and *ACAN* by performing RT-PCR [46,47,48]. These markers were significantly higher than those of undifferentiated MSCs.

Taken together, the results of this study indicate that cells derived from bone marrow fulfilled the minimal criteria of MSCs as defined by the ISCT [37]. The results of this study demonstrate that the cells isolated from the bone marrow of the forest musk deer were mesenchymal stem cells. MSCs are of considerable significance for biodiversity conservation efforts and cell therapy for forest musk deer.

## 5. Conclusions

MSCs are attractive vehicles for developing cell-based therapies and for the study of diseases. In this study, bone marrow mesenchymal stem cells were isolated from forest musk deer for the first time. We demonstrated that forest musk deer bone marrow MSCs have a high degree of similarity to MSCs of other species. The combination of RT-PCR and flow cytometry resulted in the confirmation of MSC positive expression of surface and differentiation markers. Forest musk deer bone marrow mesenchymal stem cells exhibit stemness and differentiation ability. In addition, we constructed a cDNA library of forest musk deer MSCs, thus permitting functional genomic analysis of the forest musk deer and building a reference genome. These results lay the foundation for further research to assess whether bone marrow MSCs can be a promising tool for autologous stem cell therapy and regenerative medicine in forest musk deer.

## Figures and Tables

**Table 1:** Antibody sources.

Antibody	Source	CAT#
CD105	abcam	ab11415
CD166	eBioscience	12-1668
CD73	abcam	ab157335
CD44	abcam	ab269300
CD14	eBioscience	11-0149
HLA-DR	adcam	ab1182
CD19	eBioscience	11-0199
CD34	eBioscience	11-0349
CD45	eBioscience	11-0459

## Data Availability

The datasets generated in this study can be found in National Center for Biotechnology Informatics (NCBI) BioSample Accession: SAMN15782435 (https://www.ncbi.nlm.nih.gov/biosample/?term=SAMN15782435).

## Supplementary Materials

The following supporting information can be downloaded at: https://www.mdpi.com/article/10.3390/ani13010017/s1, Table S1: Primers for qPCR.

## References

[1] Fan Z., Li W., Jin J., Cui K., Yan C., Peng C., Jian Z., Bu P., Price M., Zhang X. (2018). The draft genome sequence of forest musk deer (*Moschus berezovskii*). Gigascience.

[2] Qiu X., Guo Q., Liu X., Luo H., Fan D., Deng Y., Cui H., Lu C., Zhang G., He X. (2018). Pien Tze Huang Alleviates Relapsing-Remitting Experimental Autoimmune Encephalomyelitis Mice by Regulating Th1 and Th17 Cells. Front. Pharmacol..

[3] Tsoi B., Chen X., Gao C., Wang S., Yuen S.C., Yang D., Shen J. (2019). Neuroprotective Effects and Hepatorenal Toxicity of Angong Niuhuang Wan Against Ischemia-Reperfusion Brain Injury in Rats. Front. Pharmacol..

[4] Wijnstekers W. (2011). The Convention on International Trade in Endangered Species of Wild Fauna and Flora (CITES)—35 Years of Global Efforts to Ensure That International Trade in Wild Animals and Plants Is Legal and Sustainable. Forensic Sci. Rev..

[5] Pittenger M.F., Mackay A.M., Beck S.C., Jaiswal R.K., Douglas R., Mosca J.D., Moorman M.A., Simonetti D.W., Craig S., Marshak D.R. (1999). Multilineage Potential of Adult Human Mesenchymal Stem Cells. Science.

[6] Mushahary D., Spittler A., Kasper C., Weber V., Charwat V. (2018). Isolation, cultivation, and characterization of human mesenchymal stem cells. Cytom. Part A.

[7] Webb T.L., Quimby J.M., Dow S.W. (2012). In vitro comparison of feline bone marrow-derived and adipose tissue-derived mesenchymal stem cells. J. Feline Med. Surg..

[8] Zhuang Y., Li D., Fu J., Shi Q., Lu Y., Ju X. (2015). Comparison of biological properties of umbilical cord-derived mesenchymal stem cells from early and late passages: Immunomodulatory ability is enhanced in aged cells. Mol. Med. Rep..

[9] Woodbury D., Schwarz E.J., Prockop D.J., Black I.B. (2000). Adult rat and human bone marrow stromal cells differentiate into neurons. J. Neurosci. Res..

[10] Song Z., Cong P., Ji Q., Chen L., Nie Y., Zhao H., He Z., Chen Y. (2015). Establishment, Differentiation, Electroporation and Nuclear Transfer of Porcine Mesenchymal Stem Cells. Reprod. Domest. Anim..

[11] Luo Y., Xie L., Mohsin A., Ahmed W., Xu C., Peng Y., Hang H., Zhuang Y., Chu J., Guo M. (2019). Efficient generation of male germ-like cells derived during co-culturing of adipose-derived mesenchymal stem cells with Sertoli cells under retinoic acid and testosterone induction. Stem Cell Res. Ther..

[12] Liu Y., Liu Y., Yie S., Lan J., Pi J., Zhang Z., Huang H., Cai Z., Zhang M., Cai K. (2013). Characteristics of mesenchymal stem cells isolated from bone marrow of giant panda. Stem Cells Dev..

[13] An J.H., Li F.P., He P., Chen J.S., Cai Z.G., Liu S.R., Yue C.J., Liu Y.L., Hou R. (2020). Characteristics of Mesenchymal Stem Cells Isolated from the Bone Marrow of Red Pandas. Zoology.

[14] Enes S.R., Ahrman E., Palani A., Hallgren O., Bjermer L., Malmstrom A., Scheding S., Malmstrom J., Westergren-Thorsson G. (2017). Quantitative proteomic characterization of lung-MSC and bone marrow-MSC using DIA-mass spectrometry. Sci. Rep..

[15] Ayscue L.H., Ross D.W., Ozer H., Rao K., Gulley M.L., Dent G.A. (1990). Bcr/abl recombinant DNA analysis versus karyotype in the diagnosis and therapeutic monitoring of chronic myeloid leukemia. Am. J. Clin. Pathol..

[16] Ramos T.L., Ignacio Sanchez-Abarca L., Muntion S., Preciado S., Puig N., Lopez-Ruano G., Hernandez-Hernandez A., Redondo A., Ortega R., Rodriguez C. (2016). MSC surface markers (CD44, CD73, and CD90) can identify human MSC-derived extracellular vesicles by conventional flow cytometry. Cell Commun. Signal..

[17] Thaweesapphithak S., Tantrawatpan C., Kheolamai P., Tantikanlayaporn D., Roytrakul S., Manochantr S. (2019). Human serum enhances the proliferative capacity and immunomodulatory property of MSCs derived from human placenta and umbilical cord. Stem Cell Res. Ther..

[18] Fiorentini E., Granchi D., Leonardi E., Baldini N., Ciapetti G. (2011). Effects of osteogenic differentiation inducers on in vitro expanded adult mesenchymal stromal cells. Int. J. Artif. Organs.

[19] Götz S., García-Gómez J.M., Terol J., Williams T.D., Nagaraj S.H., Nueda M.J., Robles M., Talón M., Dopazo J., Conesa A. (2008). High-throughput functional annotation and data mining with the Blast2GO suite. Nucleic Acids Res.

[20] Fu X., Liu G., Halim A., Ju Y., Luo Q., Song G. (2019). Mesenchymal Stem Cell Migration and Tissue Repair. Cells.

[21] Zhai Y., Li W., Zhang Z., Cao Y., Wang Z., Zhang S., Li Z. (2018). Epigenetic states of donor cells significantly affect the development of somatic cell nuclear transfer (SCNT) embryos in pigs. Mol. Reprod. Dev..

[22] Tropel P., Noël D., Platet N., Legrand P., Benabid A.-L., Berger F. (2004). Isolation and characterisation of mesenchymal stem cells from adult mouse bone marrow. Exp. Cell Res..

[23] Ashton B.A., Eaglesom C.C., Bab I., Owen M.E. (1984). Distribution of fibroblastic colony-forming cells in rabbit bone marrow and assay of their osteogenic potential by an in vivo diffusion chamber method. Calcif. Tissue Int..

[24] Mohamed-Ahmed S., Fristad I., Lie S.A., Suliman S., Mustafa K., Vindenes H., Idris S.B. (2018). Adipose-derived and bone marrow mesenchymal stem cells: A donor-matched comparison. Stem Cell Res. Ther..

[25] Arnhold S.J., Goletz I., Klein H., Stumpf G., Beluche L.A., Rohde C., Addicks K., Litzke L.F. (2007). Isolation and characterization of bone marrow-derived equine mesenchymal stem cells. Am. J. Veter Res..

[26] Liu Q., Zhu Y., Qi J., Amadio P.C., Moran S.L., Gingery A., Zhao C. (2019). Isolation and characterization of turkey bone marrow-derived mesenchymal stem cells. J. Orthop. Res..

[27] Zuk P.A., Zhu M., Mizuno H., Huang J., Futrell J.W., Katz A.J., Benhaim P., Lorenz H.P., Hedrick M.H. (2001). Multilineage cells from human adipose tissue: Implications for cell-based therapies. Tissue Eng..

[28] Zuk P.A., Zhu M., Ashjian P., De Ugarte D.A., Huang J.I., Mizuno H., Alfonso Z.C., Fraser J.K., Benhaim P., Hedrick M.H. (2002). Human adipose tissue is a source of multipotent stem cells. Mol. Biol. Cell.

[29] Bieback K., Kern S., Klüter H., Eichler H. (2004). Critical parameters for the isolation of mesenchymal stem cells from umbilical cord blood. Stem Cells.

[30] In’t Anker P.S., Scherjon S.A., Kleijburg-van der Keur C., de Groot-Swings G.M., Claas F.H., Fibbe W.E., Kanhai H.H. (2004). Isolation of mesenchymal stem cells of fetal or maternal origin from human placenta. Stem Cells.

[31] Kern S., Eichler H., Stoeve J., Klüter H., Bieback K. (2006). Comparative analysis of mesenchymal stem cells from bone marrow, umbilical cord blood, or adipose tissue. Stem Cells.

[32] Chi J., Fu B., Nie W., Wang J., Graphodatsky A.S., Yang F. (2005). New insights into the karyotypic relationships of Chinese muntjac (*Muntiacus reevesi*), forest musk deer (*Moschus berezovskii*) and gayal (*Bos frontalis*). Cytogenet. Genome Res..

[33] Asumda F.Z., Chase P.B. (2011). Age-related changes in rat bone-marrow mesenchymal stem cell plasticity. BMC Cell Biol..

[34] Nunomura S., Shimada S., Kametani Y., Yamada Y., Yoshioka M., Suemizu H., Ozawa M., Itoh T., Kono A., Suzuki R. (2012). Double expression of CD34 and CD117 on bone marrow progenitors is a hallmark of the development of functional mast cell of Callithrix jacchus (common marmoset). Int. Immunol..

[35] Savic L.J., Doemel L.A., Schobert I.T., Montgomery R.R., Joshi N., Walsh J.J., Santana J., Pekurovsky V., Zhang X., Lin M. (2020). Molecular MRI of the Immuno-Metabolic Interplay in a Rabbit Liver Tumor Model: A Biomarker for Resistance Mechanisms in Tumor-targeted Therapy?. Radiology.

[36] Yang H., Wu S., Feng R., Huang J., Liu L., Liu F., Chen Y. (2017). Vitamin C plus hydrogel facilitates bone marrow stromal cell-mediated endometrium regeneration in rats. Stem Cell Res. Ther..

[37] Dominici M., Le Blanc K., Mueller I., Slaper-Cortenbach I., Marini F., Krause D., Deans R., Keating A., Prockop D., Horwitz E. (2006). Minimal criteria for defining multipotent mesenchymal stromal cells. The International Society for Cellular Therapy position statement. Cytotherapy.

[38] Russell K.A., Chow N.H.C., Dukoff D., Gibson T.W.G., LaMarre J., Bette D.H., Koch T.G. (2016). Characterization and Immunomodulatory Effects of Canine Adipose Tissue- and Bone Marrow-Derived Mesenchymal Stromal Cells. PLoS ONE.

[39] Gu W., Hong X., Le Bras A., Nowak W.N., Bhaloo S.I., Deng J., Xie Y., Hu Y., Ruan X.Z., Xu Q. (2018). Smooth muscle cells differentiated from mesenchymal stem cells are regulated by microRNAs and suitable for vascular tissue grafts. J. Biol. Chem..

[40] Venkatesh K., Sen D. (2017). Mesenchymal Stem Cells as a Source of Dopaminergic Neurons: A Potential Cell Based Therapy for Parkinson’s Disease. Curr. Stem Cell Res. Ther..

[41] Fujii Y., Fujii K., Nakano K., Tanaka Y. (2003). Crosslinking of CD44 on human osteoblastic cells upregulates ICAM-1 and VCAM-1. FEBS Lett..

[42] Takahashi N., Knudson C.B., Thankamony S., Ariyoshi W., Mellor L., Im H.J., Knudson W. (2010). Induction of CD44 cleavage in articular chondrocytes. Arthritis Rheum..

[43] Narcisi R., Cleary M.A., Brama P.A., Hoogduijn M.J., Tüysüz N., Berge D.T., van Osch G.J. (2015). Long-Term Expansion, Enhanced Chondrogenic Potential, and Suppression of Endochondral Ossification of Adult Human MSCs via WNT Signaling Modulation. Stem Cell Rep..

[44] Wang Q., Rozelle A.L., Lepus C.M., Scanzello C.R., Song J.J., Larsen D.M., Crish J.F., Bebek G., Ritter S.Y., Lindstrom T.M. (2011). Identification of a central role for complement in osteoarthritis. Nat. Med..

[45] Loeser R.F. (2014). Integrins and chondrocyte-matrix interactions in articular cartilage. Matrix Biol..

[46] Bokui N., Otani T., Igarashi K., Kaku J., Oda M., Nagaoka T., Seno M., Taternatsu K., Okajima T., Matsuzaki T. (2008). Involvement of MAPK signaling molecules and Runx2 in the NELL1-induced osteoblastic differentiation. FEBS Lett..

[47] Ambati S., Yu P., McKinney E.C., Kandasamy M.K., Hartzell D., Baile C.A., Meagher R.B. (2016). Adipocyte nuclei captured from VAT and SAT. BMC Obes..

[48] de Crombrugghe B., Lefebvre V., Behringer R.R., Bi W., Murakami S., Huang W. (2000). Transcriptional mechanisms of chondrocyte differentiation. Matrix Biol..

