# Supplementary material for: Isolation and Identification of Bone Marrow Mesenchymal Stem Cells from Forest Musk Deer
Source: Animals (Basel). 2022 Dec 20;13(1):17. doi: 10.3390/ani13010017 (PMC9817501; doi:10.3390/ani13010017)
Supplement: Supplementary file 1 [file animals-13-00017-s001.zip › animals-2032793-supplementary.pdf]

Table S1. Primers for qPCR

Gene	Sequence (5' - 3')	Length(bp)
CD105	Forward: GCGATGGCATGACTCTGGTA<br>Reverse: TCGTCTTCGGTTTCCTCAGC	112
CD44	Forward: CGCAGAACCAAGAGGACAGT<br>Reverse: CCAAGTCTCCCACCGAACTC	162
OPN	Forward: AACGTTCAGAGTCCAGGTGT<br>Reverse: GGAAAGCTCGCTACTGTTGG	132
CD29	Forward: TCTGGATACCACTTCCTGCAT<br>Reverse: AGGCAGGTCTGACACATCTC	132
CD59	Forward: GTTCTTCCTGGCTGTCCTCT<br>Reverse: GGTAAACTTGTAGCGGCACA	146
PPAR	Forward: GGTGTTTGCGAGTGTTTCTATGTG<br>Reverse: GGTTCAGTCTCCTGGAACGCC	176
ADIPOQ	Forward: TAGGTGTCAAACTCGTCGCT<br>Reverse: ATGAAGAACGCAGAGGAGGT	167
RUNX2	Forward: AGGCACAGACAGAAGCTTGA<br>Reverse: AGGACTTGGTGCAGAGTTCA	134
